# Fire deficit increases wildfire risk for many communities in the Canadian boreal forest

**DOI:** 10.1038/s41467-020-15961-y

**Published:** 2020-05-01

**Authors:** Marc-André Parisien, Quinn E. Barber, Kelvin G. Hirsch, Christopher A. Stockdale, Sandy Erni, Xianli Wang, Dominique Arseneault, Sean A. Parks

**Affiliations:** 1 Natural Resources Canada, Canadian Forest Service, Northern Forestry Centre, Edmonton, AB T6H 3E5 Canada; 2 Natural Resources Canada, Canadian Forest Service, Great Lakes Forestry Centre, Sault Ste. Marie, ON P6A 2E5 Canada; 3 Département de biologie, chimie et géographie, Université du Québec à Rimouski, Rimouski, QC G5L 3A1 Canada; 4 Aldo Leopold Wilderness Research Institute, Rocky Mountain Research Station, US Forest Service, 790 E Beckwith Ave., Missoula, MT USA

**Keywords:** Boreal ecology, Fire ecology, Environmental sciences, Natural hazards

## Abstract

The top priority of fire management agencies in Canada is to protect human life and property. Here we investigate if decades of aggressive fire suppression in the boreal biome of Canada has reduced the proportion of recently burned forests (RBF; <30 years) near human communities, and thereby inadvertently increased the risk of wildfire. We measured the percentage of RBF, which are usually less flammable than older forests, up to a 25-km radius around communities compared to that in the surrounding regional fire regime zone. Our analysis of 160 communities across boreal Canada shows that 54.4% exhibited a deficit or lack of RBF, whereas only 15.0% showed a surplus. Overall, a majority (74.4%) of communities are surrounded by a low (≤10%) proportion of RBF, indicating a higher vulnerability of those communities to wildfire. These findings suggest that suppression policies are increasing flammability in the wildland–urban interface of boreal Canada.

## Abstract (web summary)

A primary element of modern wildfire management is to aggressively suppress small fires before they become large, but benefits can be offset by the fact that these practices promote older forests that are more ‘flammable’. Here the authors show that this downside puts numerous human communities at elevated risk of fires in boreal Canada.

## Introduction

Over the past half century, Canada’s forests have experienced an upward trend in annual area burned, particularly in western Canada^1,2^. Identifying the drivers of this change is, however, challenging given that wildfire is a complex phenomenon influenced by several natural and human variables^3,4^. This includes changes in weather and climate resulting from increased atmospheric CO_2_, fuel (i.e., flammable biomass) composition and continuity, human land use, and fire management policies and practices. Increasing population growth in wildfire-prone areas^5^ combined with projections of greater burn rates due to climate change could further exacerbate the risk of wildfire to people and property^6,7^. In Canada, this growing threat of wildfire requires a comprehensive approach to fire management^8^, of which one aspect is efficient fire suppression around communities. However, the effect that management policies and practices have had on boreal burn rates is still not fully understood^9^. For example, although people are responsible for more than half of ignitions in boreal Canada^10^, people also limit wildfire through fire-suppression activities, such as early detection and rapid initial attack, resulting in a net decrease in area burned by wildfires^11,12^. Fire suppression may thus result in a wildfire deficit that has directly contributed to an increase in the amount and continuity of flammable biomass, especially around communities^13,14^.

The wildfire regimes of the North American boreal forest are characterized by a combination of numerous small fires and relatively infrequent large high-intensity crown fires that have a disproportionate impact (e.g., 3% of wildfires burn ~97% of the total area burned)^1^. Those large fires greatly reduce the amount of flammable material and are mostly lethal to trees^15^. Post-fire boreal forest stands that are <30 years in age are less likely to burn than older forests due to the combined effects of depletion of biomass after fire and the slow regrowth of vegetation (i.e., low productivity) of high-latitude forests^16^. This leads to a reduced potential for fire ignition and subsequent propagation^17^, and this negative feedback between fire and fuels has been observed throughout the biome (Supplementary Fig. 1)^17–20^. Without this feedback, burn rates (i.e., percent annual area burned) would be substantially higher^21,22^.

In Canada, fire management efforts are spatially variable and can be broadly split into ‘intensive’ and ‘extensive’ fire protection zones^23,24^. The ‘intensive’ zone is managed with aggressive and systematic fire-suppression policies due to its greater wildland–urban interface (WUI) and natural resource-related values (e.g., timber, oil, and gas). In this zone, fire management is focused on aggressive fire suppression that involves rapid detection and response to extinguish low-to-moderate intensity fires, while they are relatively small (i.e., typically less than a few hectares in size). In the more remote ‘extensive zone’, fires are often monitored but generally allowed to burn with minimal intervention, unless there are human values at risk, in which case active fire suppression is employed^25^. Fire management agencies throughout the country prioritize public safety and community protection over other values, regardless of the fire management zone^26^. In other words, while fire-suppression policies may affect fire regimes over broad areas^12,27^, their impacts are most pronounced close to communities.

Decades of aggressive fire suppression around communities in boreal Canada likely has altered the forest mosaic by promoting the retention of older forest stands compared to what would have occurred under a naturally functioning fire regime^28^. Have the long-term effects of fire management policies limited the prevalence of young stands and increased forest flammability around boreal communities? If such an increase is borne out, fires burning in the vicinity of some communities will likely be more intense (i.e., in terms of energy release) and be more difficult to control than those in the overall forest matrix^29^. Anecdotally, recent catastrophic wildfire events in the WUI have burned through forests that had been spared by wildfires for several decades. This was the case of the 2016 Fort McMurray wildfire (Alberta, Canada), which became the costliest natural disaster in modern Canadian history ($3.64 billion CND in insured losses), as this 590,000-fire spread almost entirely through forests that had not burned since the 1940s or earlier.

**Fig. 1:**
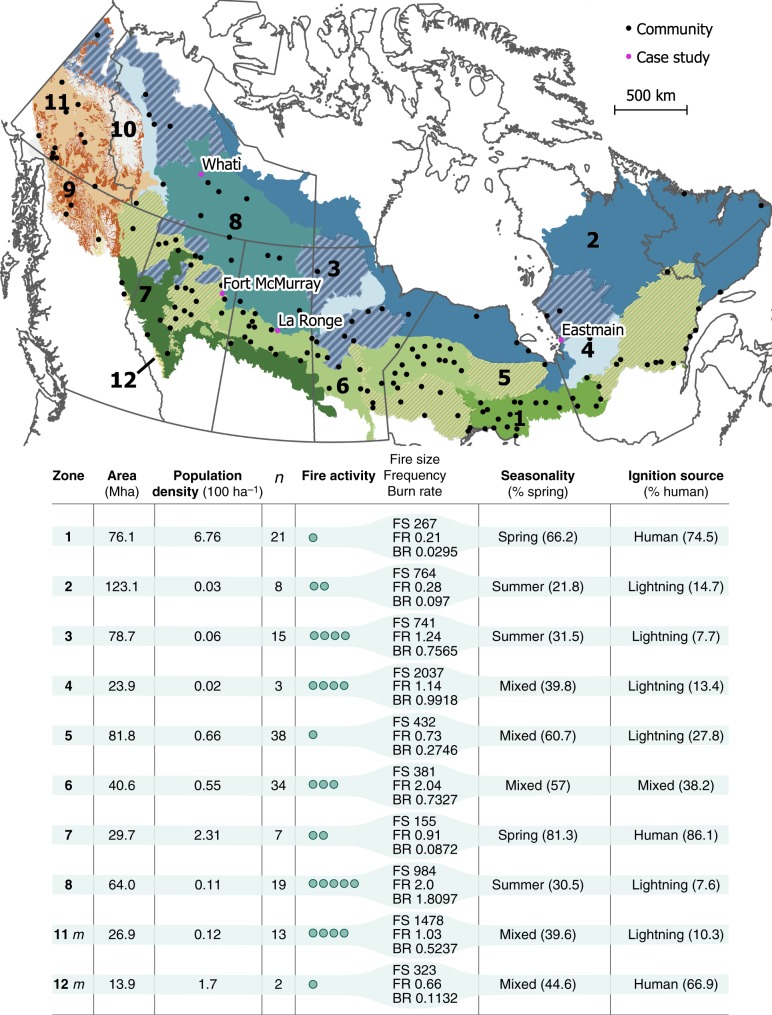
Fire regime zones in the Canadian boreal forest and the 160 studied communities. The four case study communities are named (pink dots on map). The fire activity index, rated from low to extreme (scaled from one to five dots), is a combination of fire size (FS), fire frequency (FF), and burn rate (BR)^30^. The seasonality index represents the season in which >65% of the total annual area burned: spring, summer, or mixed when no season dominates. The proportion of area burned during spring is indicated in parenthesis. The ignition source represents the type of ignition that caused >65% of the annual number of fires: human- or lightning-caused, or mixed when no cause dominates. The *m* suffix for the zones 11 and 12 refers to a mountainous topography. Note that some zones are geographically discontinuous; zones 9 and 10 are nonboreal, and therefore not considered for analysis. This figure was created using QGIS 3.10.0, under GNU General Public License v2, 1991 (gnu.org/licenses/old-licenses/gpl-2.0.en.html).

Here, we investigate the potential flammability of forest cover surrounding communities relative to the overall forested landscape in the boreal biome of Canada. Specifically, we calculated the proportion of forest stands that burned <30 years ago (termed ‘recently burned forests’ (RBF)) in 5-km radius concentric bands (up to 25-km) around selected communities and compared this to the RBF percentage within the forest matrix further away from communities that are considered to have similar fire regimes (hereafter ‘fire regime zones’ (FRZ); Fig. 1)^30^. This allowed us to: (1) compare the proportion of RBF around communities to that of the corresponding FRZ, (2) identify where high fire hazard (RBF ≤  10%) communities were located, and (3) qualitatively evaluate whether communities recently impacted by wildfire were predominantly surrounded by old forest, suggesting a fire deficit and increased risk of wildfire.

## Results

### RBF around boreal communities

**Fig. 2:**
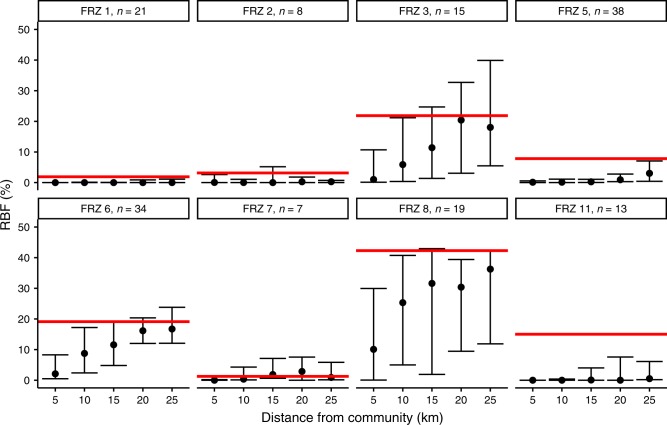
Recently burned forest around communities by fire regime zone. Percent of RBF within 5-km buffers (nonoverlapping) around communities in each fire FRZ. Points and error bars represent bootstrapped medians and 95% confidence intervals, respectively. Horizontal red lines indicate the percentage of RBF for the FRZ. FRZ with fewer than six communities are not considered. Statistical significance is inferred if the error bars do not intersect the red line.

Our results show a low percentage of RBF (forests <30 years old) around most communities (Fig. 2) and a fire deficit relative to the overall forest matrix in all FRZ (except for FRZ 7), especially in FRZ with high burn rates. Communities in FRZ 5 and 11 have a statistically significantly lower percentage of RBF at all spatial distances (5-km concentric buffers) compared to the percentage for their respective FRZ (95% bootstrap intervals). This is also the case for communities in FRZ 3 up to 10 km; FRZ 8 for 5, 10, 20, and 25 km; and FRZ 6 and FRZ 7 for the first 5 km. There are also significantly lower RBF percentages for communities in FRZ 1 and 2, but these absolute differences are minimal (<5% RBF). It is worth noting that because the 5-km buffers around communities were not overlapping, the ‘fire deficit’ is cumulative among concentric rings.

**Fig. 3:**
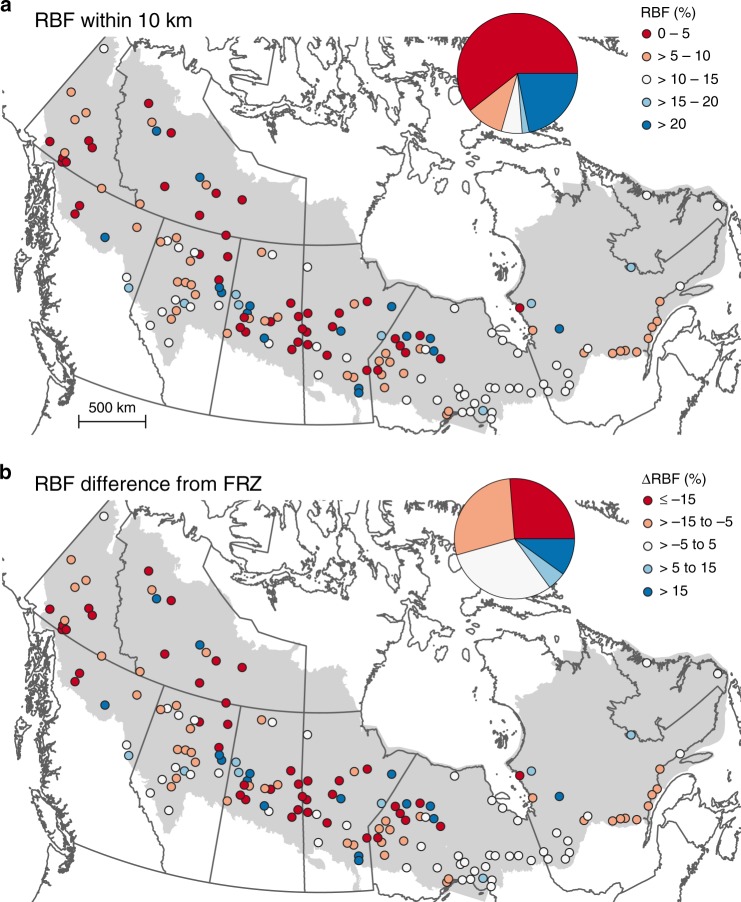
Recently burned forest and fire deficit/surplus. Percentage RBF in a 10-km buffer surrounding communities **a** and the difference of RBF around communities from the percentage of their respective FRZ **b**. Red indicates less RBF, which suggests elevated fire hazard. This figure was created using QGIS 3.10.0, under GNU General Public License v2, 1991 (gnu.org/licenses/old-licenses/gpl-2.0.en.html).

A majority (74.4%) of communities in the boreal biome of Canada are surrounded by older forests (i.e., ≤ 10% RBF) within a 10-km radius (Fig. 3a). When relativized according to the mean values of their FRZ, there is a fire deficit (defined as ≥5% RBF difference from that in the FRZ) in 54.4% of communities, a fire surplus in 15.0%, and no substantial difference in the remainder (Fig. 3b).

### Other factors affecting wildfire risk

Barriers to the spread of wildfires (e.g., water, deciduous forests, and permanent nonfuels) varied among FRZ. Communities in most FRZ (1, 3, 5, 6, 8, and 11) had a significantly (*p* < 0.05; Mann–Whitney test) greater percentage of open water in their vicinity (10-km radius) compared to randomly selected points in that zone (Supplementary Table 1). However, we found no significant correlation between the proportions of water in a 10-km radius around communities and percent RBF (Supplementary Table 2). The percent cover of deciduous forest (often associated with RBF) was generally greater for random points than communities, whereas the proportion of permanent nonfuel was virtually identical overall between the two sets of points (Supplementary Table 1). Logging is another factor that could increase young forests near communities, but the percentage of timber harvest units from 1984 to 2015 surrounding communities was generally similar to or lower than the FRZ percentage (Supplementary Fig. 2).

### Examples of recently evacuated communities

**Fig. 4:**
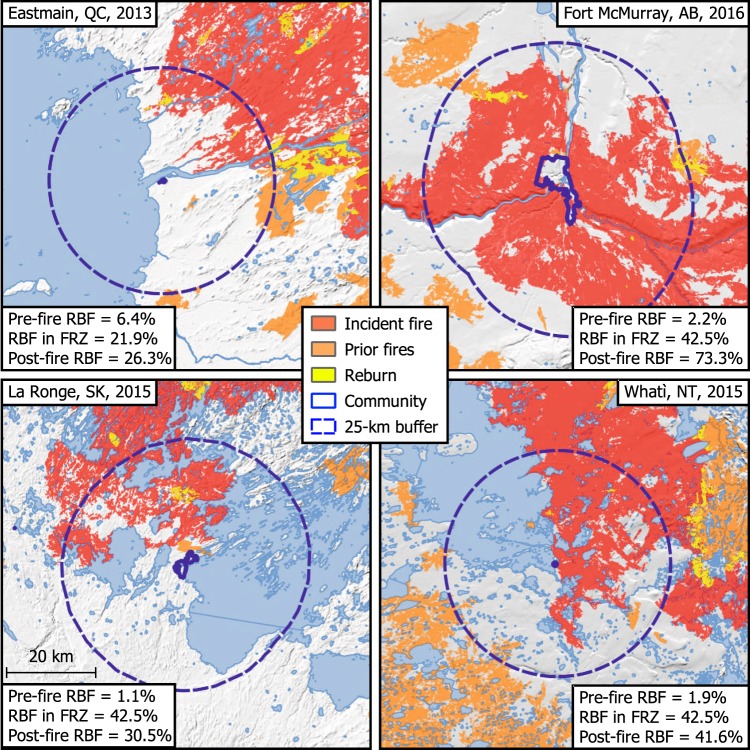
Case studies of recent large wildfires near communities. Prior fires are those within 30 years of the case study wildfire. The comparison of the pre-fire percentage of recently burned forest (pre-fire RBF) to the percentage of RBF in the fire regime zone (RBF in FRZ) indicate a fire deficit prior to the wildfire. This deficit is largely or entirely nullified by the case study wildfires (post-fire RBF). This figure was created using QGIS 3.10.0, under GNU General Public License v2, 1991 (gnu.org/licenses/old-licenses/gpl-2.0.en.html).

A qualitative assessment of four recent high-profile wildfires that burned near or into communities indicates a low pre-fire RBF abundance relative to surrounding FRZ (Fig. 4). The pre-fire percent RBF in the vicinity of Eastmain, QC was 6.4%, whereas the FRZ percentage was 21.9%; around Fort McMurray, AB, La Ronge, SK, and Whatì, NT the pre-fire RBF was 2.2%, 1.1%, and 1.9%, respectively, whereas the FRZ percentage was 42.5%. In terms of fire ignitions, an analysis revealed that the density of all ignitions is 21.6 times higher within 5 km of our sample communities than in the areas beyond this buffer, and is 62.3 times higher for human-caused ignitions (e.g., Supplementary Fig. 3). The percentage of human-caused ignitions varies from 13% to 79% among FRZ, with most human-caused wildfires concentrated within a 10-km radius around communities; there is no relationship between the density of lightning-caused fires and town proximity (Supplementary Fig. 4).

## Discussion

Wildland fires are dependent on three factors that are increasingly coinciding in time and space: flammable fuels, ignitions, and fire-conducive weather^31^. The recently observed increase in large-wildfire frequency in parts of Canada is due in part to observed increases in the fire weather severity^32^, and several studies suggest this trend could accelerate under a changing climate^6,33,34^. Further contributing to increases in the risk of wildfire is the expanding interface between communities, infrastructure, and the forest, which exacerbates human exposure to catastrophic wildfire^5^. Currently, fire suppression is the main fire management technique used in Canada with almost one billion dollars (CND) spent annually on firefighting crews, equipment, and aircrafts^35^. While suppression has proven effective in many parts of the boreal forest^11,12^, its effectiveness is likely to degrade as limited fire-suppression resources become increasingly overwhelmed under projected climate changes^36^.

Given that protecting people and property is the highest priority for fire management agencies^26^ and that most fire ignitions (per unit area) occur closest to human development, firefighting resources are often stationed in or near communities. This allows them to attack many fires when they are small and low in intensity, thus increasing the probability of successful containment^37^. Extinguishing wildfires before they become large increases the age of the forest and the amount and continuity of flammable vegetation near communities, thereby unintentionally amplifying the fire hazard in the long run around these same values that we seek to protect. In fact, successful fire suppression without subsequent modification of the forest (i.e., fuel treatments, harvesting, and other disturbances) will inevitably lead to vegetation conditions characteristic of a fire deficit. This effect, termed “fire paradox”^38^, has already been observed in many fire-prone ecosystems of the USA (refs. ^39,40^). Our results show that this is also occurring in parts of Canada.

Forest stands <30 years old are not only less flammable in terms of fire ignition and spread, but they may also provide the added benefit of creating a ‘fire shadow’ extending several kilometers on the lee side of recent burns by interrupting the spread of incoming wildfires^41^. We show that, relative to the forest matrix, there is a significant lack of RBF within 5 km and up to 25 km for many communities in the Canadian boreal biome, a majority of which are located in the western half of Canada and in FRZ where settlements are generally more remote and the burn rates are higher. However, little or no fire deficit around a community does not necessarily imply low exposure to wildfire. In eastern Canada, for example, wildfires are inherently less frequent, generally resulting in lower fire deficits surrounding communities compared to western Canada. Consequently, forests in general and those around communities in eastern Canada are considerably older than those in western Canada (Fig. 3a), and they do occasionally experience very large wildfires^42^. Also of note is that factors other than fire suppression can influence the amount of RBF. For instance, many communities may be inherently less flammable because they are located near a large water body, but our results suggest that this is not generally the case (also exemplified in Fig. 4). Although water bodies can certainly assist fire-suppression activities, Nielsen et al.^43^ showed that substantial reductions in fire likelihood are mainly restricted to areas around large lakes >5000 ha or when the proportion of water on the landscape is >40%.

Communities with low RBF are vulnerable to fast-spreading, high-intensity wildfires beyond fire control capabilities. Nowhere is this better exemplified than in the 2016 Fort McMurray wildfire, where fire suppression began in the 1940s and has been especially intensive since the 1970s. This has led to the area immediately around the community being uncommonly free from recent fires and young fuels, particularly given the fire proneness of the region^10^. Although the inhabitants of this community suffered significant emotional stress^44^ and financial losses, the probability of another large, catastrophic wildfire in the coming decades has been greatly reduced around the community because of the large prevalence of RBF. This said, occasionally wildfires can burn stands <30 years of age if the weather conditions are extreme enough^45^. This was evident in the 2013 Eastmain fire (442,510 ha) that burned several kilometers into an 8-year-old forest during 2 days of extreme weather^22^. Identifying the specific weather conditions at which the flammable biomass limitation is overcome represents an active area of research^40,46^; however, high-resolution historical reconstructions^16^ and meteorological analysis of particularly extreme fire seasons, such as the 2014 season in the Northwest Territories (Supplementary Fig. 1)^47^, suggest that the reburning of RBF remains a relatively rare event even under the most extreme of conditions.

Our results reaffirm the urgent need for a more holistic approach to wildland fire management that emphasizes proactive mitigation to complement an efficient fire-suppression system as a means to reducing the risk of wildfires to communities^8^. Examples of actions that could be taken include: enhancing fire prevention programming^48^, reducing the ignition potential of structures^49^, implementing targeted fuel (i.e., vegetation) management^50^, and, where feasible, prescribed burning^51,52^. There are, however, many physical, policy, and operational challenges to overcome. Mechanical fuel treatments require continued maintenance over time, are too expensive to apply across large boreal landscapes^53^, and are usually not designed to halt extreme wildfires^54^. Unlike the forests of other North American biomes, vegetation management in the boreal forest can be less focused on modifying forest structure (e.g., thinning and pruning) and instead emphasize changes to forest composition. For example, where possible and ecologically desirable, encouraging the establishment of deciduous stands through forestry practices represents an attractive option for drastically reducing the likelihood of wildfire over larger expanses of forest^55^.

Although prescribed burning programs in boreal forests would reset the stand age and effectively reduce wildfire potential for decades to come^56,57^, the propensity of coniferous boreal forests to burn as high-intensity crown fires can make prescribed burning a risky proposition for some residents and decision-makers. The risk tolerance to prescribed burning may, however, increase as the need for hazard reduction becomes critical. For example, in Banff, Alberta (hemi-boreal vegetation), which welcomes millions of tourists annually, Parks Canada has garnered public approval to maintain an active prescribed burning program for forests adjacent to the town since the early 1990s. The risk to the community is minimized by burning almost exclusively during the spring and fall, and by burning small parcels frequently (i.e., over several years) rather than conducting single large burns, practices that are consistent with traditional indigenous burning practices in that area^51^.

Large, high-intensity wildfires are inevitable in the boreal forest and the absence of other plausible factors explaining reductions of RBF around many communities lends credence to the presumption that fire management policies, particularly fire suppression, are contributing to a fire deficit. Identifying the critical proportion of RBF necessary to substantially reduce the risk remains an open question, but even a modest increase in the proportion of RBF could be highly beneficial in some instances, especially when fire management strategies maximize the use of non- or less-flammable features across the landscape. The disproportionately high density of ignitions around communities combined with a greater continuity of older forests in some locales in boreal Canada may increase the call for more fire suppression, especially if projections of more extreme fire weather are borne out^58^. Recognizing, however, that there is no single solution or silver bullet to reducing the risk of wildfires to communities, breaking the continuity of older, highly flammable forests must be an element of a comprehensive approach to managing wildland fire risk^26^.

## Methods

### Study area and FRZ

The study area included all of boreal Canada (Fig. 1), as defined by the ecozones of the national ecological framework for Canada^59^. This region is generally bounded to the north by the transition to tundra and to the south by prairie and temperate forest ecosystems. This study area was subdivided for analysis into 12 FRZ, ecographic units considered to have similar fire regimes^30^. Although the zonation exists for all of Canada, only those FRZ in the boreal biome were considered, and of these, zones with fewer than six communities (e.g., zones 4 and 12) were excluded from the analyses summarized by FRZ (see below). All analyses were performed using the R software v3.6.1 and ArcGIS Enterprise v10.7 for Windows.

### Selection of forest communities

From an initial dataset of 381 boreal communities, 160 communities were selected from a geographic dataset of populated places^60^ based on the following criteria. Communities with a population <200 (ref. ^61^) were excluded from the analysis because many of them were temporary (or even abandoned) settlements. We removed communities with >30% natural or anthropogenic nonfuel within a 25-km buffer (e.g., barren and agriculture) to eliminate boreal communities with largely non-forested surroundings. Communities on islands in large lakes were also excluded. Next, we retained only one community from pairs or groups within 25 km of one another to eliminate spatial overlap in the analysis. A community was retained if its population was at least twice that of these nearby communities. If this condition was not met, the community with the greatest forest cover within 10 km was retained, based on land cover analysis of 2014 forest vegetation^62^. Finally, the remaining 160 community centroids were spatially corrected using aerial imagery (Bing Maps, https://www.bing.com/maps/aerial).

### Wildfire and timber harvesting data

The National Burned Area Composite was used to analyze wildfire patterns across the study area^63^. Fire polygons were rasterized at a resolution of 250 m and overlaid to calculate time since fire. All permanent nonfuel (i.e., tundra, water, and open rock) was masked out using the kNN Canadian land cover dataset^62^. We produced a map of RBF by classifying any pixel with an age <30 years (1988–2017) as RBF, and any pixel with an age ≥30 years as non-RBF. Forest harvest areas, which were based on a Canada-wide Landsat-based 30-m resolution change-detection dataset from 1985–2015 (ref. ^64^; see below), were not considered in the calculation of RBF.

### RBF around boreal communities

Statistics on RBF were computed for each community and FRZ. Concentric buffers, 5 km in width and centered on community boundaries, were used as analysis units, up to a maximum of 25 km. We calculated the percent RBF of the total area of forested lands within buffers after masking permanent nonfuels (e.g., open water). To limit any interaction among neighboring communities, parts of buffers were removed where they overlapped another community’s buffer with a narrower radius (e.g., a 20–25 km buffer overlapping a neighboring community’s 15–20 km buffer). We removed all pixels within 2 km of community boundaries to limit the effects of outlying residences, infrastructure, and other compounding factors. The percentage of RBF within these concentric buffers, averaged for all communities of a FRZ, was compared against the percentage of RBF of the entire FRZ in which they were located. The analysis was also performed using an alternative fire zonation scheme, the homogeneous fire zones (HFZ)^34^, for comparison; although the results were broadly similar, HFZ were too large and encompassed an unacceptable degree of variation in fire regimes, and were thus subsequently dropped.

### Sensitivity analysis

The 30-year cutoff for RBF was based on the published studies conducted in the Canadian boreal forest^16,45^. To investigate the sensitivity of this threshold, we repeated the analysis with a threshold of 20 (1998–2017) and 40 (1978–2017) years. The 20- and 40-year definitions of RBF yielded results coherent to those of the 30-year definition, suggesting that our results are not overly sensitive to the choice of age threshold (Supplementary Fig. 5). Despite spatial variation in forest types and ecological processes across boreal Canada, the 30-year-old threshold for RBF was robust throughout the biome.

### Ignitions and other factors affecting wildfire risk

We conducted several supplementary analyses to investigate whether other factors were related to the prevalence of RBF near communities, including ignition density, forest harvest percentage, and land cover. Ignition density was calculated within 5-km concentric buffers centered on our selected communities and compared to the ignition density outside of this buffered area, considering all fires larger than 0.1 ha from 1988–2017. We also verified if forest harvest from 1985–2015 (ref. ^64^) was disproportionately higher around our selected communities relative to the areas of the fire zones. Using the 5-km buffers, we compared the percentage area harvested near communities against percentage area harvested across FRZ for a 30-year period.

Finally, to examine whether land cover surrounding populated areas was qualitatively different than those of their respective FRZ, we compared the proportion of water, permanent nonfuel (excluding water), and deciduous forest, which is considered substantially less flammable than coniferous forests. The analysis of water was carried out using the Canada MODIS Land Cover Time Series^65^ and nonfuel and deciduous forest cover was calculated from the National Risk Analysis Fuel map^66^, both of which have a 250-m resolution.

### Reporting summary

Further information on research design is available in the Nature Research Reporting Summary linked to this article.

## Supplementary information

Supplementary Information

Reporting Summary

## Data Availability

The datasets generated during or analysed during this study area available at the Centre for Open Science OSF data repository [https://osf.io/kqp2y/]. Source data underlying Figs. 2 and 3, Supplementary Figs. 2, 4, and 5, and Supplementary Tables 1 and 2 are provided in a Source Data file in the OSF data repository.
