# Author Correction: Fire deficit increases wildfire risk for many communities in the Canadian boreal forest

**DOI:** 10.1038/s41467-020-17650-2

**Published:** 2020-07-28

**Authors:** Marc-André Parisien, Quinn E. Barber, Kelvin G. Hirsch, Christopher A. Stockdale, Sandy Erni, Xianli Wang, Dominique Arseneault, Sean A. Parks

**Affiliations:** 1 Natural Resources Canada, Canadian Forest Service, Northern Forestry Centre, Edmonton, AB T6H 3E5 Canada; 2 Natural Resources Canada, Canadian Forest Service, Great Lakes Forestry Centre, Sault Ste. Marie, ON P6A 2E5 Canada; 3 Département de biologie, chimie et géographie, Université du Québec à Rimouski, Rimouski, QC G5L 3A1 Canada; 4 Aldo Leopold Wilderness Research Institute, Rocky Mountain Research Station, US Forest Service, 790 E Beckwith Ave., Missoula, MT USA

**Keywords:** Boreal ecology, Fire ecology, Environmental sciences, Natural hazards

Correction to: *Nature Communications* 10.1038/s41467-020-15961-y, published online 1 May 2020.

The original version of this article contained an error in Fig. 3, in which panel **a** was duplicated in panel **b**. The correct version of Fig. 3 is:



which replaces the previous incorrect version:



This has been corrected in both the PDF and HTML versions of the Article.

