# Supplementary material for: Fire deficit increases wildfire risk for many communities in the Canadian boreal forest
Source: Nat Commun. 2020 May 1;11:2121. doi: 10.1038/s41467-020-15961-y (PMC7195457; doi:10.1038/s41467-020-15961-y)
Supplement: Supplementary file 1 — Supplementary Information [file 41467_2020_15961_MOESM1_ESM.pdf]

Supplementary Information - Parisien et al., Fire deficit increases wildfire risk for many communities in the Canadian boreal forest

Authors

Marc-André Parisien[1,a]
Quinn E. Barber[1]
Kelvin G. Hirsch[1,*]
Christopher A. Stockdale[1]
Sandy Erni[2]
Xianli Wang[2]
Dominique Arseneault[3]
Sean A. Parks[4]

Author affiliations

[1]Natural Resources Canada, Canadian Forest Service, Northern Forestry Centre, Edmonton AB, T6H 3E5
[2]Natural Resources Canada, Canadian Forest Service, Great Lakes Forestry Centre, Sault Ste. Marie, ON, P6A 2E5
[3]Département de biologie, chimie et géographie, Université du Québec à Rimouski, Rimouski, QC, G5L 3A1
[4]Aldo Leopold Wilderness Research Institute, Rocky Mountain Research Station, US Forest Service, 790 E. Beckwith Ave., Missoula, MT, USA.
[*]Retired

[a]Correspondence and requests for materials should be addressed to M.-A.P. (email: marc-andre.parisien@canada.ca)

Supplementary Figure 1. Wildfires of two recent extreme fire seasons, 2014 and 2015, in the southern Northwest Territories, Canada. Wildfires (red outlines) are underlain by fires that occurred with 30 years prior to the year of interest (2014 [left] and 2015 [right]). Note that the extent of most of the 2014 and 2015 wildfires is limited by prior fires. Fire perimeters are from the National Burned Area Composite (NBAC)[1]. This figure was created using QGIS 3.10.0, under GNU General Public License v2, 1991 (gnu.org/licenses/old-licenses/gpl-2.0.en.html).

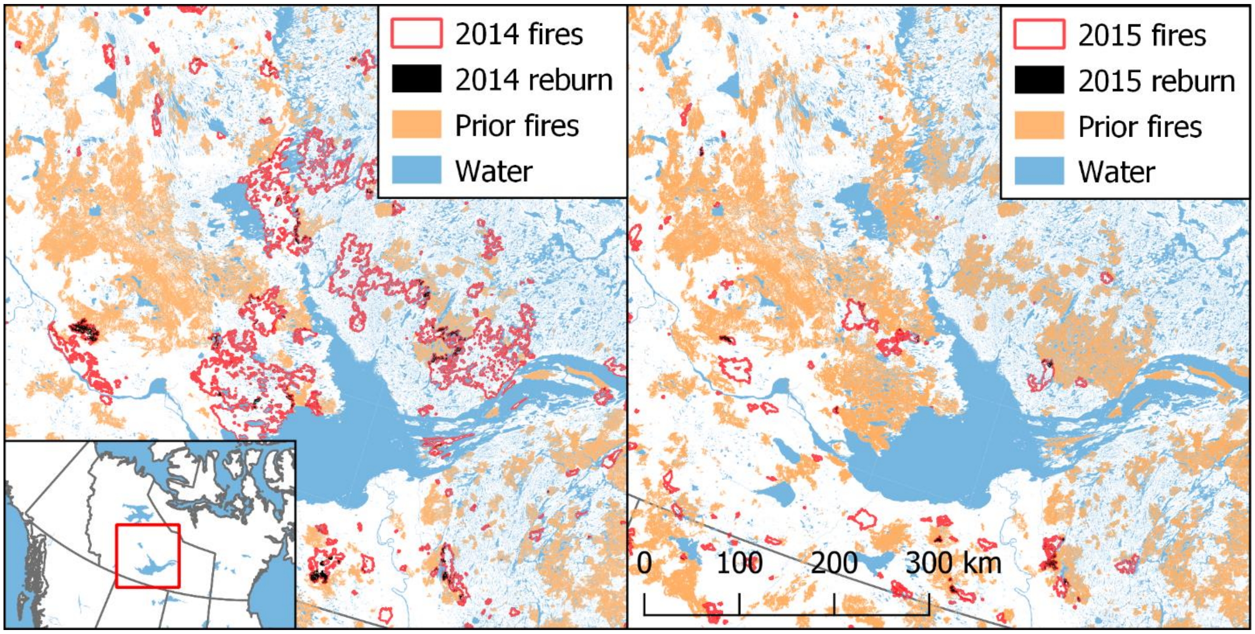

Supplementary Figure 2. Forest harvesting by fire regime zone (FRZ). Percent of harvested patches were calculated within 5-km buffers (non-overlapping) around communities in each FRZ. Points and error bars represent bootstrapped medians and 95% confidence intervals, respectively. Horizontal red lines indicate proportion of forest harvesting for the FRZ. FRZ with less than six communities are not considered. Statistical significance is inferred if the error bars do not intersect the red line. Harvest area is calculated from Canada-wide Landsat-based 30-m resolution change-detection dataset from 1985-2015[2].

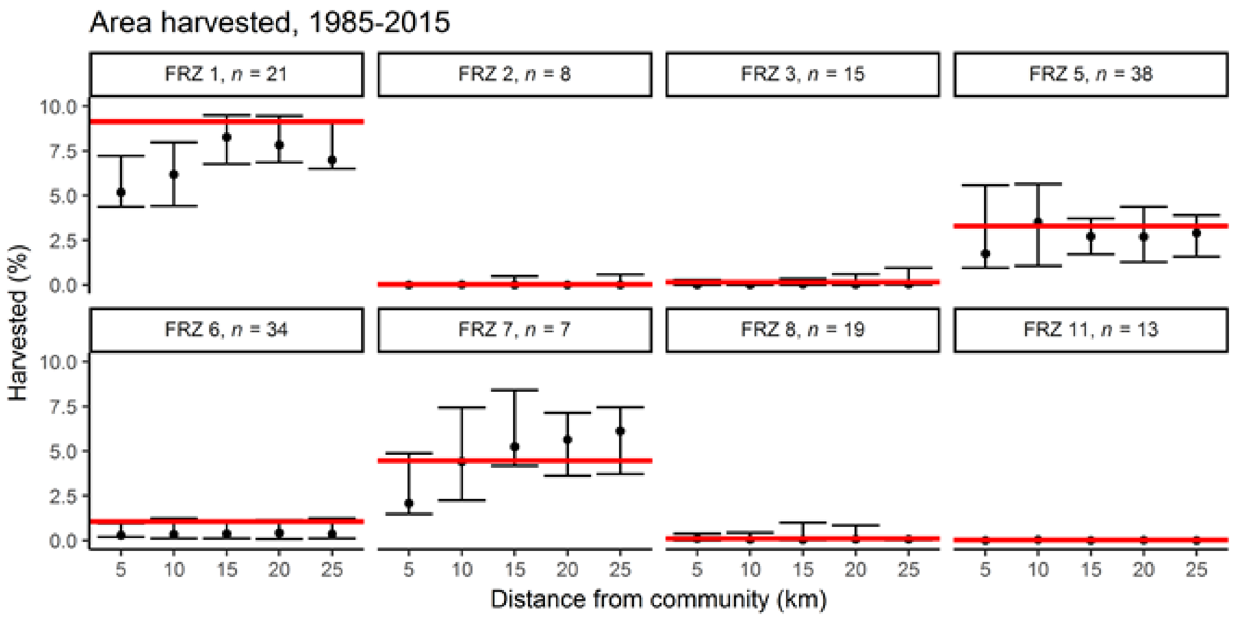

Supplementary Figure 3. Wildfire ignitions around communities. Case studies of recent ignitions near communities, based on the Canadian National Fire Database[3]. All ignitions from fires greater than 0.1 ha that started in the 30 years prior to the large wildfire of interest are included. This figure was created using QGIS 3.10.0, under GNU General Public License v2, 1991 (gnu.org/licenses/old-licenses/gpl-2.0.en.html).

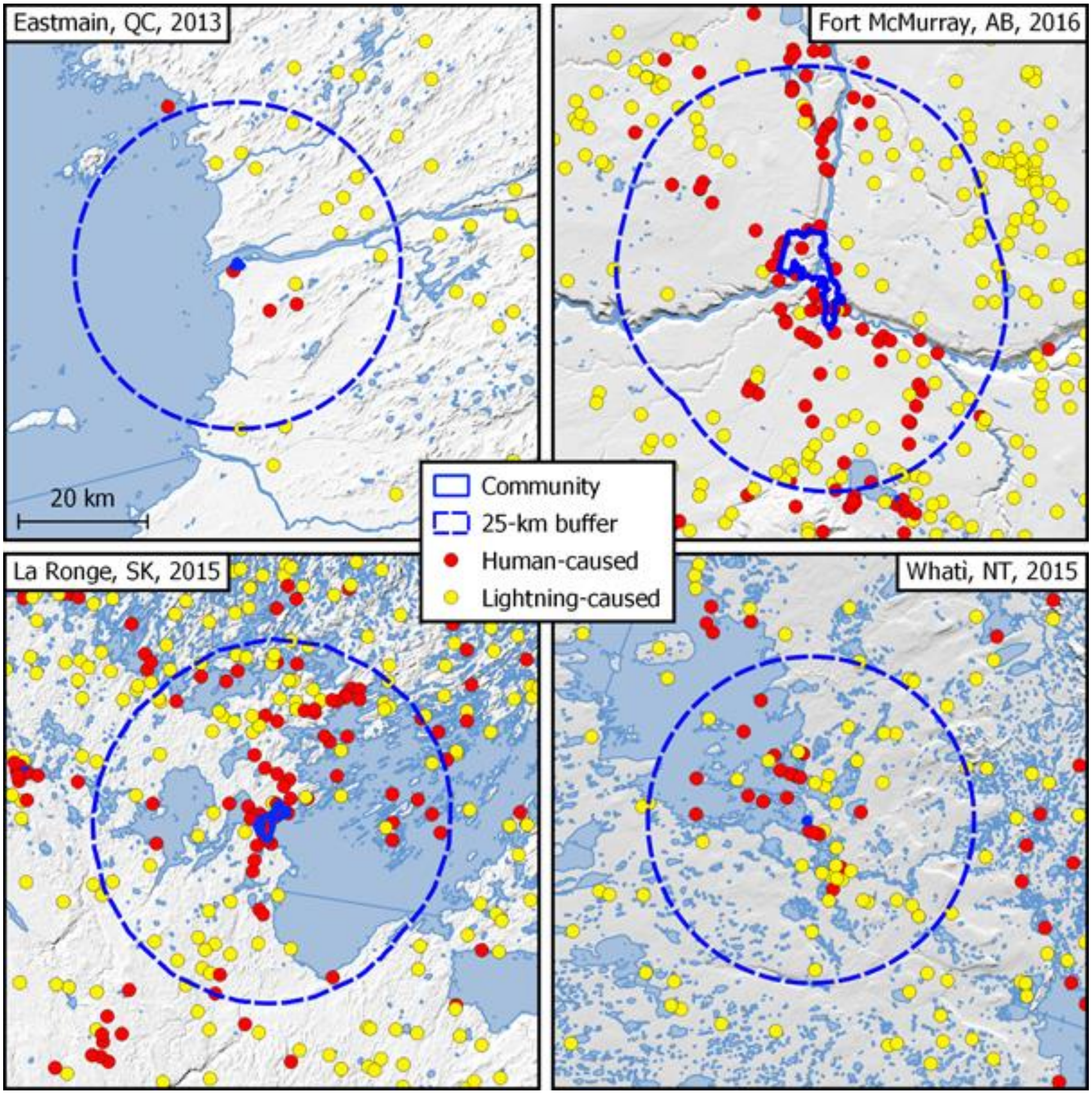

Supplementary Figure 4. Wildfire ignitions by cause in each fire regime zone (FRZ). Number of fires >0.1 ha within 5-km buffers (non-overlapping) around communities in each FRZ for the 1988-2017 period, based on the Canadian National Fire Database[3]: (a) the density of all fire ignitions (human and lightning); (b) the density of human-caused ignitions; (c) the density of lightning-caused ignitions; and, (d) the proportion of human-caused ignitions. Points and error bars represent bootstrapped medians and 95% confidence intervals, respectively. Horizontal red lines indicate the density (a, b, c) or percentage (d) for the FRZ. FRZ with less than six communities are not considered. Statistical significance is inferred if the error bars do not intersect the red line. These results show that: (1) human-caused wildfires are numerous, but that their proportion varies by zones; (2) human-caused wildfires are generally concentrated within a 10-km radius around communities; and, (3) there is no relationship between the density of lightning-caused fires and town proximity.

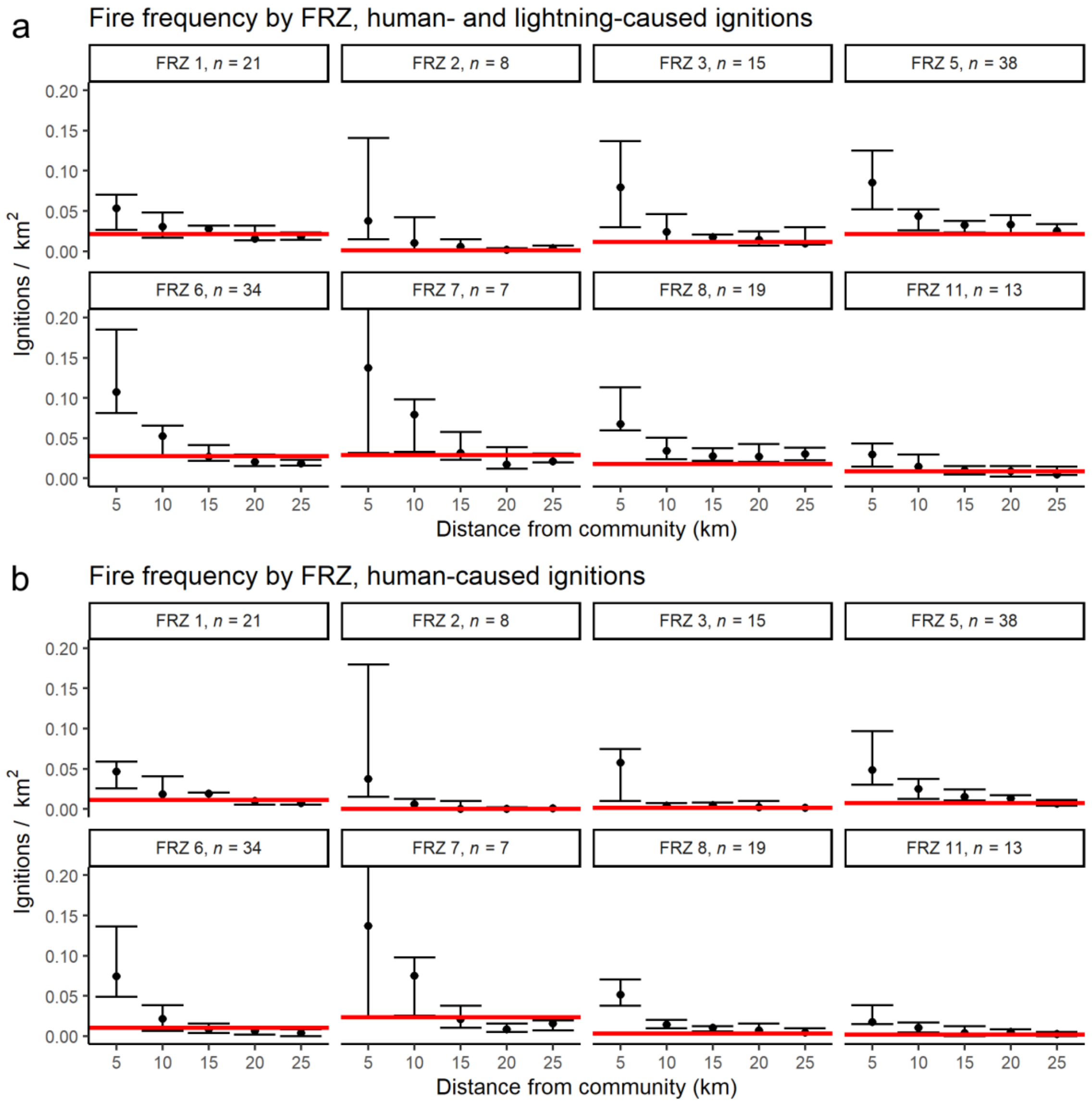

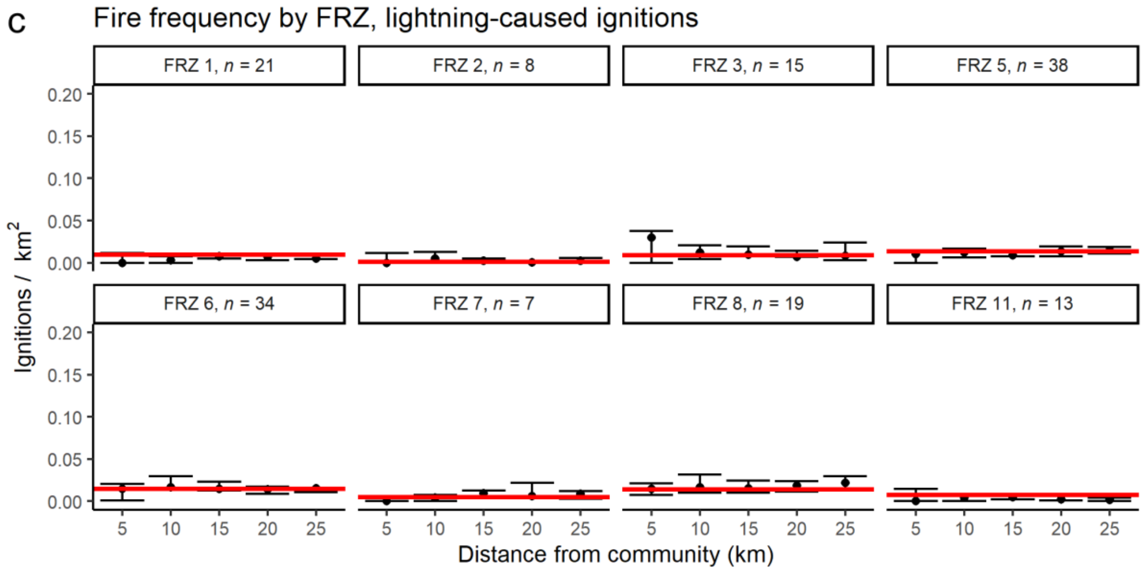

c
Fire frequency by FRZ, lightning-caused ignitions
FRZ 1, n = 21
FRZ 2, n = 8
FRZ 3, n = 15
FRZ 5, n = 38
FRZ 6, n = 34
FRZ 7, n = 7
FRZ 8, n = 19
FRZ 11, n = 13
Ignitions / km2
0.00
0.05
0.10
0.15
0.20
5
10
15
20
25
Distance from community (km)

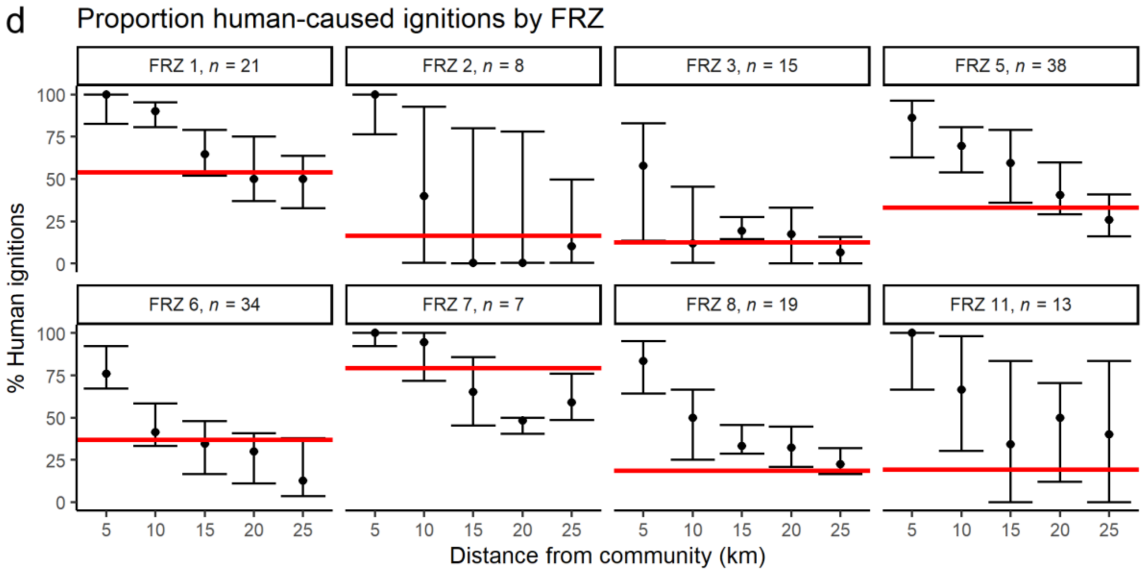

d
Proportion human-caused ignitions by FRZ
FRZ 1, n = 21
FRZ 2, n = 8
FRZ 3, n = 15
FRZ 5, n = 38
FRZ 6, n = 34
FRZ 7, n = 7
FRZ 8, n = 19
FRZ 11, n = 13
% Human ignitions
0
25
50
75
100
5
10
15
20
25
Distance from community (km)

Supplementary Figure 5. Sensitivity analysis of recently burned forest (RBF) age by fire regime zone (FRZ). Replication of results of Figure 2 for different definitions of RBF: (a) RBF < 40 years (1978-2017), and (b) RBF < 20 years (1998-2017). Percent of RBF within 5-km buffers (non-overlapping) around communities in each FRZ. Points and error bars represent bootstrapped medians and 95% confidence intervals, respectively. Horizontal red lines indicate proportion of RBF for the FRZ. FRZ with less than six communities are not considered. Statistical significance is inferred if the error bars do not intersect the red line.

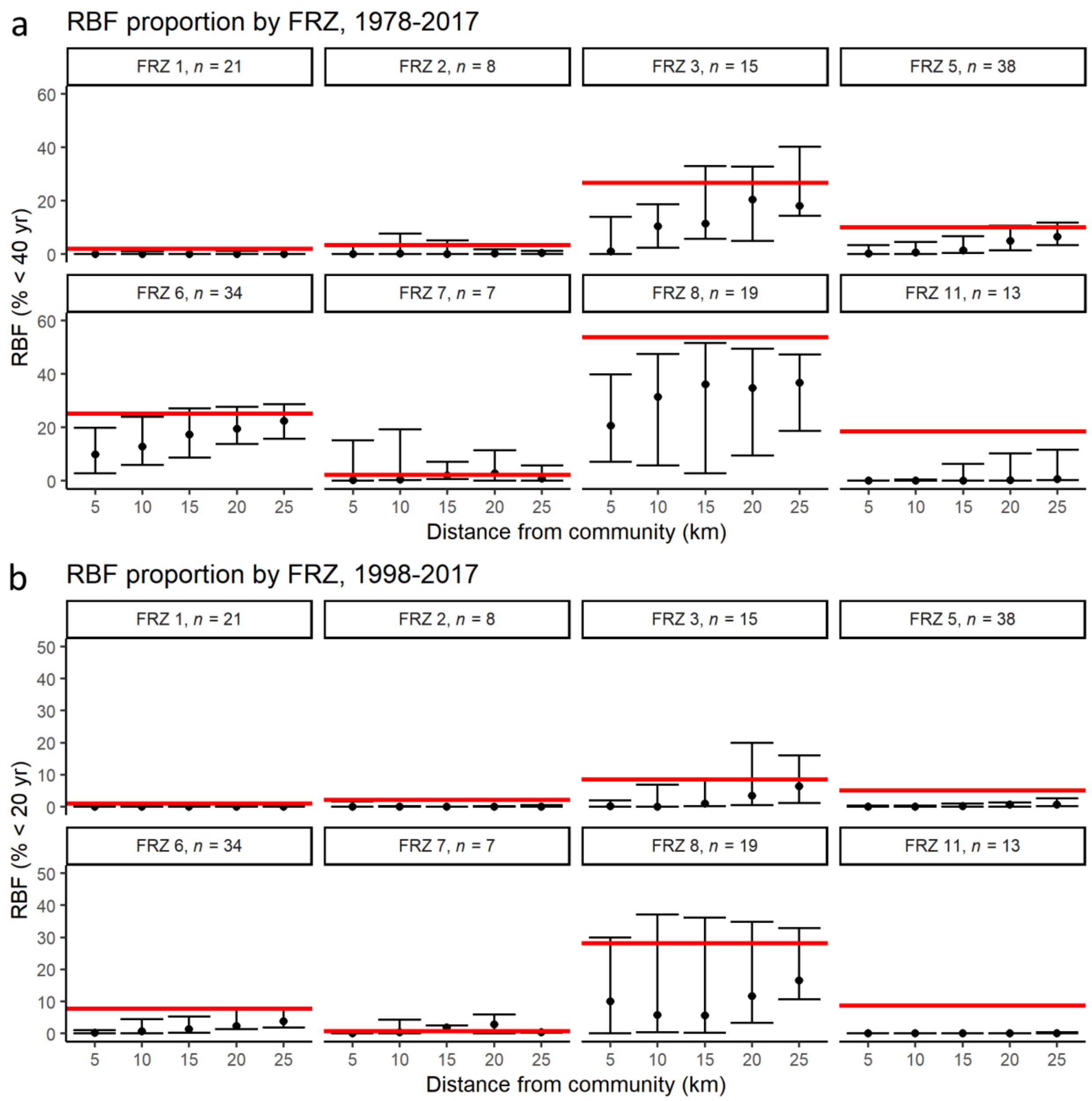

Supplementary Table 1. The percent water, deciduous forest, and nonfuel (excluding water) within a 10-km radius of the communities (C) and corresponding spatially random points (R) by FRZ and for the total study area (Total). Land cover calculations were done using the Canada MODIS Land Cover Time Series[4] for water and the National Risk Analysis Fuel map[5] for nonfuel and deciduous forest cover. Note that a 2-km internal buffer was excluded around populated places to be consistent with the study's main analysis. The sample size is denoted by *n*. The *p*-values correspond to a two-tailed Mann-Whitney test.

	n		**Water (%)**			**Deciduous (%)**			**Nonfuel (%)**		
**FRZ**	**C**	**R**	**C**	**R**	***p*-value**	**C**	**R**	***p*-value**	**C**	**R**	***p*-value**
1	21	42	11.6	5.5	0.01	6.6	5.4	0.72	3.3	2.8	0.02
2	8	275	18.0	12.6	0.52	24.4	38.6	0.19	2.2	5.6	0.01
3	15	159	26.2	12.2	0.00	18.1	27.1	0.16	3.4	4.7	0.80
5	38	153	16.0	7.7	0.00	18.2	16.2	0.50	3.2	2.2	0.01
6	34	69	26.2	12.2	0.00	18.3	20.0	0.55	4.5	2.9	0.00
7	7	35	4.6	1.6	0.83	31.3	22.3	0.20	5.1	9.1	0.66
8	19	120	25.4	12.8	0.00	14.6	24.5	0.03	10.4	5.7	0.94
11	13	56	5.2	2.2	0.01	34.8	39.2	0.72	6.6	5.2	0.02
**Total**	**155**	**1000**	**18.17**	**9.90**		**18.59**	**26.88**		**4.62**	**4.97**	

Supplementary Table 2. Spearman correlations between the percent water and the percent recently burned forest, excluding nonfuel, calculated on a 10-km buffer around points and communities (based on data from Table A1). Note: significance of rho values (p < 0.05) for the random points of FRZ 2 (rho = -0.282) and 8 (rho = --0.217) is inflated due to large sample size.

	Communities			Random points		
**FRZ**	***n***	**rho**	***p*-value**	***n***	**rho**	***p*-value**
1	21	-0.081	0.726	42	0.137	0.386
2	8	-0.168	0.691	275	-0.282	<0.000
3	15	-0.050	0.863	159	-0.055	0.518
5	38	0.002	0.993	153	-0.051	0.507
6	34	-0.046	0.795	69	-0.087	0.435
7	7	-0.321	0.498	35	0.164	0.444
8	19	-0.266	0.271	120	-0.217	0.012
11	13	-0.084	0.786	56	0.014	0.910

References

[1]Canadian Forest Service. 2019. National Burned Area Composite (NBAC). Natural Resources Canada, Canadian Forest Service, Northern Forestry Centre, Edmonton, Alberta. http://cwfis.cfs.nrcan.gc.ca.

[2]Guindon, L., P. Bernier, S. Gauthier, G. Stinson, P. Villemaire, and A. Beaudoin. 2018. Missing forest cover gains in boreal forests explained. Ecosphere 9:e02094.

[3]Canadian Forest Service. 2019. Canadian National Fire Database – Agency Fire Data. Natural Resources Canada, Canadian Forest Service, Northern Forestry Centre, Edmonton, Alberta.

[4]Natural Resources Canada. 2012. Canada 250m land cover time series 2000-2011. Government of Canada, Natural Resources Canada, Earth Science sector. Canada Centre for Remoting Sensing. Ottawa, Ontario, Canada
https://open.canada.ca/data/en/dataset/39518dfa-bb8d-8a04-b36b-50b4310527a2

[5]Natural Resources Canada. 2019. National Risk Analysis Fuels Map. Government of Canada, Natural Resources Canada, Canadian Forest Service. Northern Forestry Centre. Edmonton, Alberta, Canada
http://cwfis.cfs.nrcan.gc.ca/downloads/fuels/development/National_Risk_Analysis_Fuels_Map/
