# Supplementary material for: Fire deficit increases wildfire risk for many communities in the Canadian boreal forest
Source: Nat Commun. 2020 May 1;11:2121. doi: 10.1038/s41467-020-15961-y (PMC7195457; doi:10.1038/s41467-020-15961-y)
Supplement: Supplementary file 2 — Reporting Summary [file 41467_2020_15961_MOESM2_ESM.pdf]

nature research

Corresponding author(s): Marc-André Parisien

Last updated by author(s): Mar 30, 2020

Reporting Summary

Nature Research wishes to improve the reproducibility of the work that we publish. This form provides structure for consistency and transparency in reporting. For further information on Nature Research policies, see Authors & Referees and the Editorial Policy Checklist.

Statistics

For all statistical analyses, confirm that the following items are present in the figure legend, table legend, main text, or Methods section.

n/a	Confirmed	
☐	☒	The exact sample size (*n*) for each experimental group/condition, given as a discrete number and unit of measurement
☒	☐	A statement on whether measurements were taken from distinct samples or whether the same sample was measured repeatedly
☐	☒	The statistical test(s) used AND whether they are one- or two-sided<br>*Only common tests should be described solely by name; describe more complex techniques in the Methods section.*
☐	☒	A description of all covariates tested
☐	☒	A description of any assumptions or corrections, such as tests of normality and adjustment for multiple comparisons
☐	☒	A full description of the statistical parameters including central tendency (e.g. means) or other basic estimates (e.g. regression coefficient) AND variation (e.g. standard deviation) or associated estimates of uncertainty (e.g. confidence intervals)
☐	☒	For null hypothesis testing, the test statistic (e.g. *F*, *t*, *r*) with confidence intervals, effect sizes, degrees of freedom and *P* value noted<br>*Give P values as exact values whenever suitable.*
☒	☐	For Bayesian analysis, information on the choice of priors and Markov chain Monte Carlo settings
☒	☐	For hierarchical and complex designs, identification of the appropriate level for tests and full reporting of outcomes
☐	☒	Estimates of effect sizes (e.g. Cohen's *d*, Pearson's *r*), indicating how they were calculated

Our web collection on statistics for biologists contains articles on many of the points above.

Software and code

Policy information about availability of computer code

Data collection	No software was used specifically for data collection.
Data analysis	The R software, v3.6.1 for Windows; ArcGIS Enterprise v10.7 for Windows; custom code in R

For manuscripts utilizing custom algorithms or software that are central to the research but not yet described in published literature, software must be made available to editors/reviewers. We strongly encourage code deposition in a community repository (e.g. GitHub). See the Nature Research guidelines for submitting code & software for further information.

Data

Policy information about availability of data

All manuscripts must include a data availability statement. This statement should provide the following information, where applicable:

- Accession codes, unique identifiers, or web links for publicly available datasets
- A list of figures that have associated raw data
- A description of any restrictions on data availability

Databases used:

Note: All of these datasets are available on an OSF project file repository (https://osf.io/kqp2y/).

Canadian National Fire Database--Canadian Forest Service. Canadian National Fire Database – Agency Fire Data. Natural Resources Canada, Canadian Forest Service, Northern Forestry Centre, Edmonton, Alberta, Canada (2019).
<http://cwfis.cfs.nrcan.gc.ca/datamart>

Ecological zones of Canada--Ecological Stratification Working Group. A national ecological framework for Canada. Agriculture and Agri-Food Canada and Environment Canada, Research Branch, Centre for Land and Biological Resources Research, and Environment Canada, State of the Environment Directorate,

Ecozone Analysis Branch (1995).
<http://sis.agr.gc.ca/cansis/nsdb/ecostrat/gis_data.html>

Fire-regime zones of Canada--Erni S, et al. Developing a two-level fire regime zonation system for Canada. Can. J. For. Res. 50, 259-273 (2020) <data available upon request from the Canadian Forest Service>

Vegetation land cover--Beaudoin A, et al. Mapping attributes of Canada's forests at moderate resolution through kNN and MODIS imagery. Can. J. For. Res. 44, 521-532 (2014).
<data available upon request from the Canadian Forest Service>

Mapped communities and dwellings--Natural Resources Canada. North American Atlas – Populated Places. Dataset. Natural Resources Canada, Mapping Information Branch, The Atlas of Canada (2010).
<http://ftp.geogratis.gc.ca/pub/nrcan_rncan/vector/framework_cadre/North_America_Atlas10M/popplaces/>

Population data--Statistics Canada. A national overview - population and dwelling counts. 2001 Census: data products. Ottawa, Ontario, Canada (2002).
<https://www12.statcan.gc.ca/English/census01/products/standard/popdwell/tables.cfm>

Canada Landsat Disturbance (CanLAD)--Guindon L, Bernier P, Gauthier S, Stinson G, Villemaire P, Beaudoin A. Missing forest cover gains in boreal forests explained. Ecosphere 9, e02094 (2018).
<https://open.canada.ca/data/en/dataset/add1346b-f632-4eb9-a83d-a662b38655ad>

Flammable vegetation classifications (i.e., fuels)--Natural Resources Canada. National Risk Analysis Fuels Map. Natural Resources Canada, Canadian Forest Service, Northern Forestry Centre, Edmonton, Alberta, Canada (2019).
<data available upon request from the Canadian Forest Service>

Bing Maps
<https://www.bing.com/maps/aerial>

Figures that have associated raw data:

Figure 1: Fire-regime zones
Figure 3: Locations of communities
Figure 4: Canadian National Fire Database

Restrictions on data availability:

There are no restrictions on data availability.

Field-specific reporting

Please select the one below that is the best fit for your research. If you are not sure, read the appropriate sections before making your selection.

☐ Life sciences ☐ Behavioural & social sciences ☒ Ecological, evolutionary & environmental sciences

For a reference copy of the document with all sections, see nature.com/documents/nr-reporting-summary-flat.pdf

Ecological, evolutionary & environmental sciences study design

All studies must disclose on these points even when the disclosure is negative.

Study description	We investigate the potential flammability of forest cover surrounding communities relative to the overall forested landscape in the boreal biome of Canada. Specifically, we calculated the proportion of forest stands that burned <30 years ago (termed 'recently burned forests' [RBF]) in a 25-km radius around selected communities and compared it to the proportion within geographic units further removed from communities that are considered to have similar fire regimes. Our results demonstrate that forest ≥30 years old is the dominant land cover around most communities and therefore a substantial fire deficit exists relative to the overall forest matrix. These findings imply that fire-management policies and practices may have contributed to an increased flammability of the landscape around the very features (i.e., communities) they are aiming to protect.
Research sample	Mapped wildfire perimeters were obtained from the Canadian National Fire Dataset. All nonfuel (i.e. tundra, water, open rock) was masked out using the Canadian National Fuels Grid. We produced a map of 'recently burned forests' (RBF) by classifying any pixel with an age of 30 years or less (1988-2017) as RBF, and any pixel with an age greater than 30 years as non-RBF. Statistics on RBF were calculated for each community and FRZ. We calculated the proportion (%) RBF of the total area of forested lands within the buffer after masking nonforest. Concentric buffers, 5 km in width and centered on community boundaries, were used as analysis units, with a maximum buffer of 25 km. We removed all pixels within 2-km of a community boundaries to limit the effects of outlying residences, infrastructure, and other compounding factors. Percentage of RBF within these concentric buffers was compared against mean RBF computed the entire FRZ in which they were located, thereby contrasting the 'natural' forest age for each FRZ against the forest age near communities.
Sampling strategy	From an initial dataset of 381 communities in the boreal biome of Canada, 160 communities were selected from a geographic populated places dataset based on a set of criteria. Communities with a population less than 200 were excluded from the analysis because many of them were temporary (or even abandoned) settlements. Next, we retained only one community from pairs or groups within 25 km of one another to reduce redundancy in the analysis. When such a group or pair of communities were identified,

the largest of the communities was retained if its population was at least twice of all other communities. If this condition was not met, the community with greater forest cover was retained, based on land cover analysis 32. If these communities had similar (±10%) forest cover, the community with a greater population was retained. From this short list of communities, we removed communities with >30% natural or anthropogenic nonfuel within a 25-km buffer (e.g., barren, agriculture).

Data collection	We did not collect any of the data. All of the data sources used in this study were published and/or publicly available. Please see the list of data sources.
Timing and spatial scale	All data used in this study originated from existing datasets. The primary data, the dataset of mapped wildfires, ranged from 1978-2017., though the primary analysis used data from 1988-2017. The data on populated places consisted of national census data from 2001 and the mapped communities and dwellings in 2011. The forest harvest data spanned the 1984-2015; these data were used for exploration purposes and did not contribute to the primary analysis. Similarly, forest vegetation data, which was also used for secondary analyses, was collected through remote sensing in 2010 period, but is updated annually using mapped anthropogenic and natural disturbances.<br>Datasets are periodically updated an may vary from the version provided here. Versions available here were last accessed: Oct. 2018 (NBAC, Bing Maps), June 7, 2019 (NFDB, 2014 Fuels, 2011 MODIS Land Cover), Dec. 2018 (2019 Fuels), and Nov. 2018 (Ecozones, Fire Regime Zones, and Community shapefiles).
Data exclusions	No data was excluded from the analyses; however, a sample of study units (i.e., populated places) was selected in order to avoid redundancy and limit the spatial dependence between units. This selection was achieved according to a pre-determined rule set that appears in the Methods.
Reproducibility	The analysis is be straightforward to reproduce. All of the data is from published and/or publicly available sources. The software for analysis are common (R and ArcGIS software), the data manipulation simple, and the statistical analysis documented. All of the data will be made available.
Randomization	Randomization was not relevant in our study because, given the study's goal and the nature of the analysis, it was necessary to select study units according to a predefined set of rules.
Blinding	Blinding was not relevant in our study because we did not use human subjects as part of the experiment and people did not conduct any of the work in the field.

Did the study involve field work? ☐ Yes ☒ No

Reporting for specific materials, systems and methods

We require information from authors about some types of materials, experimental systems and methods used in many studies. Here, indicate whether each material, system or method listed is relevant to your study. If you are not sure if a list item applies to your research, read the appropriate section before selecting a response.

Materials & experimental systems

n/a	Involved in the study
☒	☐ Antibodies
☒	☐ Eukaryotic cell lines
☒	☐ Palaeontology
☒	☐ Animals and other organisms
☒	☐ Human research participants
☒	☐ Clinical data

Methods

n/a	Involved in the study
☒	☐ ChIP-seq
☒	☐ Flow cytometry
☒	☐ MRI-based neuroimaging
